# Governing in the Anthropocene: are there cyber-systemic antidotes to the malaise of modern governance?

**DOI:** 10.1007/s11625-018-0570-5

**Published:** 2018-05-14

**Authors:** Ray Ison, Jason Alexandra, Phil Wallis

**Affiliations:** 1 ASTiP (Applied Systems Thinking in Practice Group), School of Engineering and Innovation, The Open University (UK), Walton Hall, MK7 6AA UK; 2 RMIT, The School of Global, Urban and Social Studies, Melbourne, 3001 Australia; 3 Formerly Victorian Catchment Management Council, Melbourne, Australia

**Keywords:** Public policy failure, Systemic governance, Wicked problems, Praxis, Institutional innovation

## Abstract

**Abstract:**

The Anthropocene imposes new challenges for governments, demanding capabilities for dealing with complexity and uncertainty. In this paper we examine how effective governing of social-biophysical dynamics is constrained by current processes and systems of government. Framing choices and structural determinants combine to create governance deficits in multiple domains, particularly in relation to the governing of complex larger-scale social–biophysical systems. Attempts to build capability for governing ‘wicked problems’ are relevant to sustainability science and Anthropocene governance, but these have mostly failed to become institutionalised. Two cases studies are reported to elucidate how the systemic dynamics of governing operate and fail in relation to espoused purpose. In the UK attempts to enact ‘joined-up’ government’ during the years of New Labour government reveal systemic flaws and consistent praxis failures. From Australia we report on water governance reforms with implications for a wide range of complex policy issues. We conclude that innovations are needed to build capacity for governing the unfolding surprises and inherent uncertainties of the Anthropocene. These include institutionalising, or structural incorporation, of cyber-systemic thinking/practices that can also enhance empowerment and creativity that underpins sustainability science.

**Graphical abstract:**

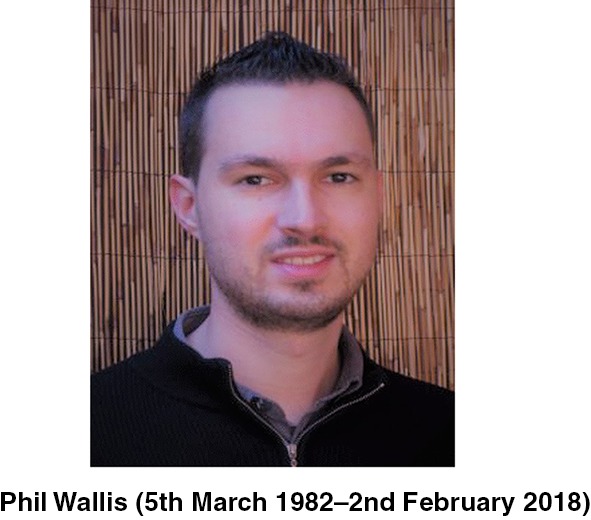

## Introduction

Given the scale, significance and magnitude of the policy challenges arising in the Anthropocene it seems necessary to take radical governance “design turns”. To design transformative governance arrangements suited to Anthropocene challenges we need to understand the institutionalised processes generating our experienced world. Such understandings are needed to inform the design of appropriate public policy and governance processes. In this paper we examine, from a mainly cyber-systemic theoretical and methodological position, the implementation of selected public sector reforms in Australia and the UK. We do so to better understand what constrains policies and their implementation from dealing effectively with Anthropocene type challenges.

The paper is structured in the following way: After this introduction, the second section deals with defining the emerging Anthropocene governance deficit and outlining theories of cyber-systemic governing. These are followed by the exploration of two cases of attempted governance reforms. The penultimate section discusses the implications of our inquiry, in terms of governance innovation that may offer antidotes to prevailing paradigms, and explores some implications for sustainability science. The final section offers our main conclusions.

## Governing complexity in the Anthropocene

### A growing governance deficit

Evidence from numerous countries indicates that many “governance systems” are not fit for purpose under contemporary circumstances (Straw 2014; Kelly 2014; Ringen 2014a, b; Micklethwait and Wooldridge 2014; Tingle 2015; Johnson 2015). Symptoms of governance deficit vary across policy domains and scales (from local to the global), occurring within nations, organisations and multilateral programs (Ison and Schlindwein 2015). Reasons are debated, with Micklethwait and Wooldridge (2014) claiming that, “for 500 years, the West’s ability to reinvent the state has enabled it to lead the world. Today, the West is weighed down by dysfunctional governments, bloated budgets and self-indulgent publics; it risks losing its edge to more autocratic Asian states.” These authors’ contestable perspective is that the neo-liberal experiments of the post-World-War-2 (WW2) era have not proceeded far enough. In contrast, Tingle (2015) observes that these experiments resulted in, “a growing loss of institutional memory about how things have come about, and, more importantly perhaps, why they did. Without memory, there is no context or continuity for current decisions.” For Tingle (ibid) the reasons for governance failure are largely institutional, related to the enacting of governance, best understood as theory-informed practice, or praxis (Ison 2017).

Systemic failures in the UK’s system of governance are revealed by Ringen’s (2009) research. Examining New Labour’s achievements from 1997 to 2007, in terms of their social policy objectives, he found they had achieved ‘absolutely nothing’ in their flagship policies of child poverty, education, social justice and health. These findings highlight problems that emerge when governments adopt command-and-control approaches that fail to mobilise citizens or stakeholders in policy development and implementation. His sobering conclusion was that no UK government, of any political persuasion, could get done what it is elected to do. Ringen (2009) identifies deep-seated issues in the ‘system of governance’ that need revitalisation and innovation including strengthening modes of horizontal governance (Ison 2010; Phillips 2004).

Likewise, Straw (2014) argues that in the UK the present system ‘stands in the way of successful government’. Recognising that incremental changes are unlikely to work he proposes a ‘Treaty for Government’ to reinvent British governance enabling systems thinking capabilities to revitalise institutions and practices that deliver effective governance (Straw ibid). Likewise, the Australian Public Service Commission (APSC) documented persistent public policy failures—endemic indigenous disadvantage, chronic health problems like obesity, stalled water reforms, and limited responses to climate change—defined as ‘wicked problems’ that provide evidence of the need for systemic reforms. These require ‘broad recognition and understanding, including from governments and Ministers, that there are no quick fixes and simple solutions’ (APSC 2007a, p. iii). The APSC (2007a) recommended that:

“critically tackling wicked problems... calls for high levels of systems thinking [that] helps policy makers to make the connections between the multiple causes and interdependencies of wicked problems that are necessary in order to avoid a narrow approach and the artificial taming of wicked problems. Agencies need to look for ways of developing or obtaining this range of skills. (p. 33)”

With the growing complexities that arise from recognition that humans (though not all humans) are changing whole earth dynamics there are pressing needs to understand and design transformative governance regimes. Examining efforts to adopt cyber-systemic approaches to complex policies is relevant to the challenges inherent in the Anthropocene (including sustainability science).

### Governing in the Anthropocene, Econocene or Capitalocene

The Anthropocene is a neologism invented by Crutzen and Stoermer (2000), a naming response to phenomena like anthropogenic perturbations to the cycling of elements such as carbon, nitrogen, and phosphorus that are changing the chemical composition of the atmosphere, oceans and soils. The lively global discourse on the Anthropocene is occurring despite the administering body for formally naming it—the International Geological Congress (IGC)—not agreeing to its use, although in August 2016 the International Commission on Stratigraphy recommended to the IGC that ‘the Anthropocene needs to be declared ... The new epoch should begin about 1950…’ (Guardian 2016).

Kunkel (2017), reviewing three books on the Anthropocene (Davies 2016; Moore 2015; Malm 2015), argues that the Anthropocene “expresses, first, an awareness that environmental change of the most durable significance is taking place as we speak, with unaccustomed speed ... Second, the Anthropocene gathers all disparate environmental issues under a single heading, from global warming down to the emissions of a trash incinerator … it takes in the sixth extinction as a whole as well as the starvation of sea lions off California, as fishermen with bills to pay deplete the stocks of sardine on which the sea lions depend” (p. 22). The Anthropocene condenses ‘into a single word … a gripping and intuitive story about human influences on the planet’ (Davies 2016). Kunkel also draws on American law academic Jedediah Purdy who said: ‘The Anthropocene has to be named before people can try to take responsibility for it’.

But not all agree. Kunkel (2017) points out, “Two of the most formidable contributions so far to the literature of the Anthropocene come from authors who reject the term.” Moore (2015) and Malm (2015) “have overlapping criticisms of what Moore calls ‘the Anthropocene argument’. Its defect, as Moore sees it, is to present humanity as a ‘homogeneous acting unit’, when in fact human beings are never to be found in a generic state. They exist only in particular historical forms of society, defined by distinct regimes of social property relations that imply different dispositions towards ‘extra-human nature’.” Moore proposes that the Anthropocene be renamed the ‘Capitalocene’, “since ‘the rise of capitalism after 1450 marked a turning point in the history of humanity’s relation with the rest of nature, greater than any watershed since the rise of agriculture’” (Kunkel 2017). Norgaard (2015), working in this intellectual territory for some time, favours the term ‘Econocene’ which starts much later—just after WW2 with the rise of a particularly rapacious form of capitalism, justified as ‘virtuous’ by various ideologies like monetarism (Thompson 1981).

More important than a debate about naming the Anthropocene are effective transformative responses. However, each term has revealing and concealing features as well as theoretical entailments (Ison 2016a). For example, attributing geological force to humans reveals the thermodynamic impacts of our activities but may conceal political disempowerment and further embed a sense of hopelessness and despair—neither of which contributes to achieving transformative governance. Neither Moore (2015) nor Malm’s (2015) books are political strategy but both assume “that a new and better ecological regime can come about in the twenty-first century” (Kunkel 2017). Yet such an ecological regime requires purposeful responses that are systemically desirable, politically and culturally feasible and ethically responsible. In the next sections we invite consideration of what and how we seek to govern in the ‘Anthropocene’.

### Governing what?

Concepts like the Anthropocene, Capitalocene or Econocene invite attention to intricate relationships (coupling) between humans and ‘nature’ or between social systems and biophysical systems (including other species and the physical world). Accepting these intricate relationships assists in breaking away from the common trap of seeing ‘the environment’ as something external to, and distinct from humans and framing humans as outside, rather than within the ecological sphere.

This relational perspective is articulated clearly by Pope Francis (The Holy See 2015) in his Encyclical letter *‘Laudato si’* which makes the compelling point that nothing is indifferent to humans, appealing that “with global environmental deterioration, I wish to address every person living on this planet” because “Given the scale of change, it is no longer possible to find a specific, discrete answer for each part of the problem. It is essential to seek comprehensive solutions which consider the interactions within natural systems themselves and with social systems… We are faced not with two separate crises, one environmental and the other social, but rather with one complex crisis which is both social and environmental … When we speak of the “environment”, what we really mean is a relationship existing between nature and the society … Nature cannot be regarded as something separate from ourselves or as a mere setting in which we live. We are part of nature, included in it and thus in constant interaction with it.”

The Pope’s encyclical calls for transformation of the relational dynamics between social and biophysical systems requiring systemic sensibility, combined with systems literacy (Ison and Shelley 2016). Effective Anthropocene responses mean placing these two intrinsically inter-related systems (social and biophysical) into a new co-evolutionary trajectory based on clear understanding about what is to be governed and how governing function.^1^

**Fig. 1:**
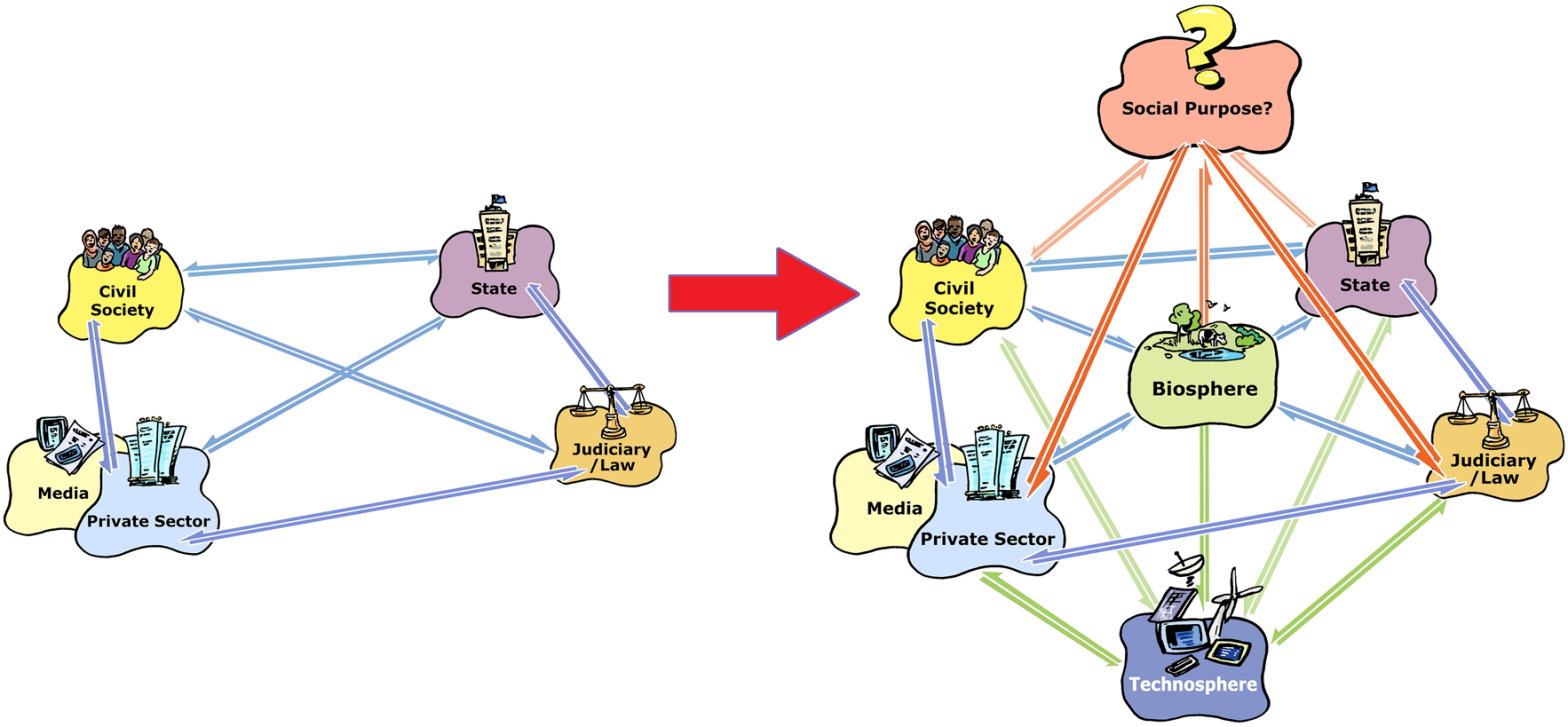
A simple ‘governance diamond with two-dimensional sets of relationships (**a** left) compared to a three-dimensional governance diamond (**b** right) needed for governing in the Anthropocene. (Source: Adapted from Ison 2016a)

Figure 1a is a heuristic for exploring the contemporary two-dimensional ‘governance diamond’ (Ison 2016a) comprising the main elements of a typical governance system with relationships between the state (civil service, the executive, ministries etc.), civil society (families, NGOs, charities etc.), the private sector (companies, SMEs, multinationals etc.) and the judiciary/courts/law. In this depiction the media is within the private sector but through burgeoning social media it is dispersing through all elements.

The governance configuration in 1b, if enacted, would provide strong, foundations for governing including defining explicit social purpose in relation to the biosphere. However, most extant governance institutions developed before humans accepted responsibility for the Anthropocene. Formal environmental management efforts are recent additions to the institutionalised regimes of most modern states. Further, current governance arrangements are poor at explicitly negotiating and pursuing social purpose because this function has been outsourced and ‘globalised’—overtaken by multinationals and global financial flows due to the veneration and reification of ‘markets’ as the source of legitimacy for the modern state (Foucault 2008). In addition, technology (the technosphere in Fig. 1) mediates governance praxis with blurring distinctions between artefactual and ‘soft’, or social, technologies. For example, it makes sense to see ‘institutions’, in the institutional economics sense, as forms of social technology (Ison 2017).

In summary, we define what we set out to govern as dynamic, systemic, relationships. Therefore governing is fundamentally relational.

### Governing how?

Governance is an “elusive and much debated concept” (Griffin 2010, p. 365). For example, policy discourses related to water and river catchments have moved from focusing on integrated management to governance (Head 2009) yet one does not replace the other. Governance is a significant expansion, broader than management, encompassing the totality of mechanisms and instruments available for directing and influencing society, including the entire cycles of adaptive planning, designing, regulating, legislating, budgeting and managing. Governance is not an abstraction; it is something that is done, enacted in theory informed and context specific ways that embed ideologies and power relations (Stirling 2012).

**Fig. 2:**
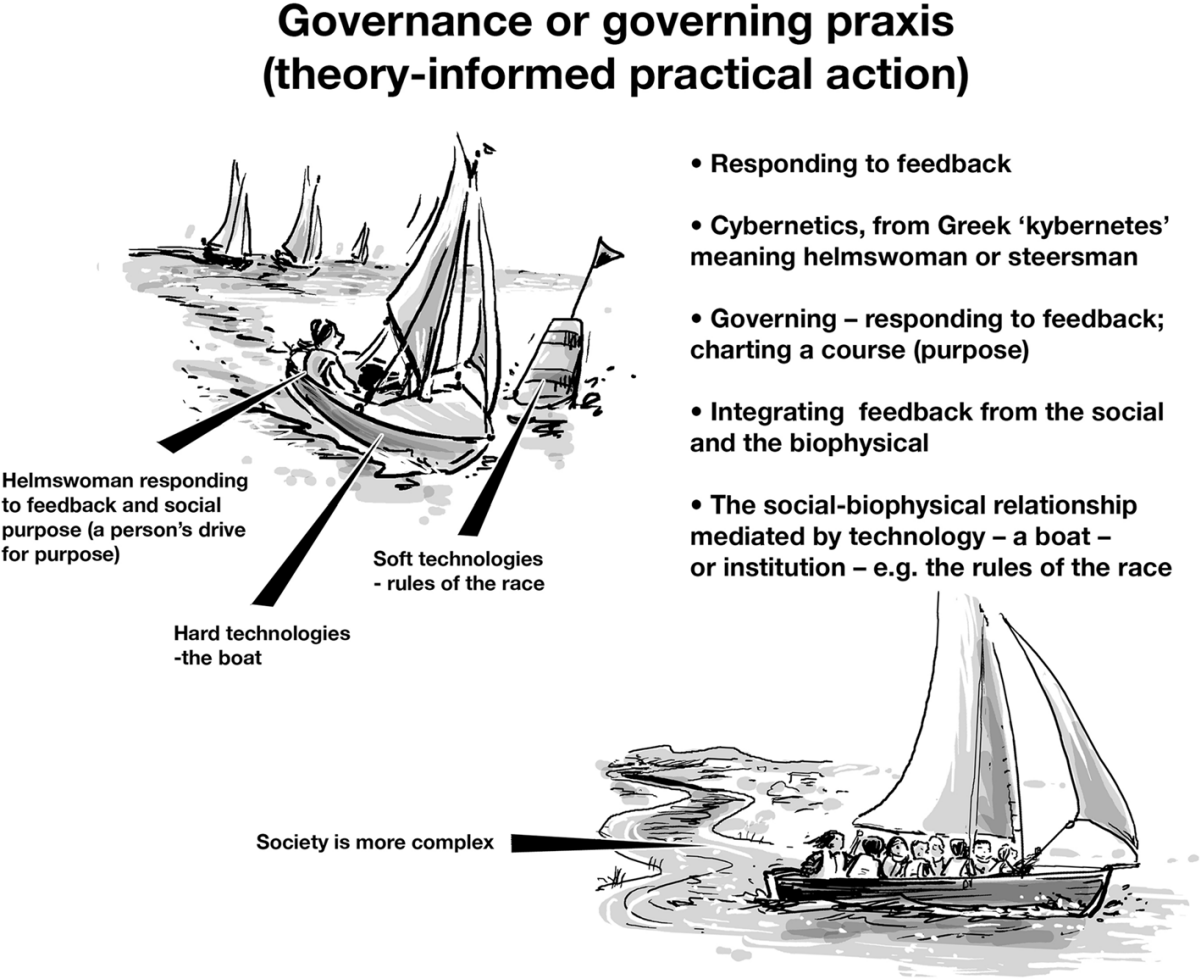
A metaphor for the praxis of cyber-systemic governing based on the Greek verbs *kybernao*/*kybernan* meaning ‘I steer/to steer’

Governing innovations are needed. Traditional institutions are failing to respond to large-scale environmental problems, like climate change that transcend established political domains (Griffin 2010). Unfortunately little recent scholarship about governance retains the nuances of its etymological roots particularly the Greek verbs *kybernao* meaning ‘I steer’ and *kybernan* meaning ‘to steer’ (i.e., the infinitive form). Ampere (1834) drew on the Greek for steering to formulate the science of civil government (see Tsien 1954). From these roots Wiener (1948) reformulated the term cybernetics, naming a field of study, which turned ‘steering’ into the science of steering, and through this labelling created the noun. With this paper we invite a return to the active verb form(s) as a basis for governance praxis. By drawing upon the intellectual lineage of cyber-systemics (Blunden and Dando 1994; Ison 2010; Ison et al. 2014a; Rhodes 1996) we frame governance using the central metaphor of a helmsperson (sailor) steering, or charting a viable course in response to feedback (from currents, wind) in relation to purposes that are renegotiated within an unfolding context—that is, in repeatedly recalibrated responses to uncertainty. The dynamics, between social and biophysical systems are mediated by artefactual technologies—such as the boat—and social technologies—like the rules of a sailing race (Fig. 2). From this metaphor we take the term ‘cyber-systemic governance/governing’. We avoid the idea that purpose, or goals, are pre-given preferring instead the idea that ‘purpose elaborating’ is integral to governing (Checkland 1985).

Finally, it is important to define the terms ‘systematic’ and ‘systemic’ that have particular meanings in cyber-systems theory (Ison 2017). Systematic approaches use linear, step-by-step thinking and action, whereas systemic ones are holistic comprising relationally dynamic thinking and acting. Systematic approaches dominate modern governments, who generally adopt linear causality, codified in hierarchical organisational structures with their routines and practices that embed managerial and ‘engineering’ type approaches. The dominance of these approaches may partially explain the governance deficits referred to above. We explore this further below.

## Investigating cases of governance reform

### Discourse coalitions and structural coupling

Having clarified our framing choices via two heuristics designed to explore governance dynamics we now explore two cases of governance reforms drawing on theories of discourse coalitions (Hajer 1995) and structure determined systems (Maturana and Varela 1987).

The first case explores the Blair-Brown government’s efforts at ‘joined-up government’ and ‘targets-focused deliverology’. In the second we explore Australian reforms intended to resolve stalled water policy reforms that APSC defined as a ‘wicked’ problem (APSC 2007a). Both cases are relevant to sustainability science because they exemplify how certain practices and understandings are conserved, and reproduced institutionally, despite attempts at systemic innovations (Ison 2016b). Both cases examine constraints to discourse coalitions (Hajer 1995) that attempted to institutionalise cyber-systemic governance capabilities. Constraints deemed to be institutional inertia can be partially understood in terms of structural coupling and the functioning of ‘structure determined systems’, concepts coined by Maturana and Varela (1987). In using these concepts we employ a mode of inquiry that asks what might be revealed or concealed by considering governance situations ***as if*** they were structure determined systems and (ii) that the ongoing structural coupling of social and biophysical systems are what requires governing into the future.^2^

Different theoretical frameworks can be used to elucidate the dynamics of governance. We draw upon Hajer’s (1995) ‘discourse coalitions’ and Maturana’s theories of structural coupling and co-evolution (Maturana and Varela 1987). Discourse coalitions are characterised by practitioners whose ideas become elements of political practice moulded *“because of political ideology or choices for a particular organisational form”* (Hajer and Dassen 2014, p. 20). Practice (or praxis) is central to how discourse coalitions operate (Hajer 1995, 2009). It constitutes *“*an ensemble of notions, ideas, concepts, and categorisations through which meaning is allocated to social and physical phenomena, and which is produced and reproduced*”* (Hajer 2009, pp. 59–60).

Since Rittel and Webber (1973) coined the terms ‘wicked’ and ‘tame’ problems there have been various attempts to forge ‘discourse coalitions’ (Hajer 1995) around building systemic responses to ‘wicked’ problems. We hypothesise that attempts to build enduring discourse coalitions have mostly failed because they do not institutionalise ideas, concepts and language with routines or practices capable of reinventing dynamic governance relationships (see Fig. 1). This occurs because determinants embedded in the structures and processes of governments limit the necessary practices and capabilities, despite these being repeatedly recognised as critically needed (e.g. APSC 2003, [Bibr CR6][Bibr CR7], 2013). Defining situations as tame, wicked, or diabolical, are framing choices (Ison et al. 2014a; Rittel and Webber 1973) with significant implications for policy development (Isendahl et al. 2010; Schön and Rein 1994) as are the way certain discourses and their logics become established.

Ringen’s (2009) research described earlier is relevant to sustainability science because it demonstrates that governments can function like structure-determined system’s with power to constrain certain discourses and institutional innovations (Maturana and Poerksen 2004; Maturana and Verden-Zoller 2008). We propose that it is impossible for some ‘discourse coalitions’ to develop and flourish in governance regimes inimical to the constellations of certain ideas, language and practices, while other discourses take root and become legitimised within and across governance regimes, institutionalising pathways that limit future options (Marshall and Alexandra 2016). For example, consider how economic rationalism became established as the dominant policy paradigm within Australia (Pusey 1991) whilst ecologically sustainable development (ESD) policies have withered (Dovers and Wild River 2002; Mercer 1998)—evidence that ‘new public management’ has proven inimical to the consolidation of alternative discourses (Chapman 2002; MacDermott 2008).

The institutions humans invent, with norms, beliefs, rules and policies, determine what can and can’t be done (Bromley 2006). The internal workings of governments are heavily institutionalised, structured by explicit rules and implicit ideologies; thus governance as enacted can be understood as like a structure-determined system, with emergent behaviour produced by what the system allows. As Maturana observes, when we seek a mechanic to fix a car we treat the car as a structure-determined system; i.e., systems operate according to how they are made through the operations of their components (Maturana and Verden-Zoller 2008, p. 158). Examples of structural determinants of governance systems include the 3-year election cycle in Australia and the UK’s first-past-the post voting system. Each helps determine what is, and is not, possible. Whilst structure determinism is inescapable, greater awareness of the systemic affordances of structure-determined systems is needed. This includes awareness of the degree to which the system is open to change through external perturbations or whether ‘destructive interactions’ may lead to the loss of a system’s current structure.

Most contemporary governance structures appear inimical to transforming situations that sustain wicked problems (Fig. 1b). But in the Anthropocene the focus of concern, is how the interacting social and a biophysical system co-evolve. This relational dynamic can be understood through the lens of structural coupling of the two systems (a social system and a biophysical system). Structural coupling happens when two or more systems in recursive interactions defined by the properties of their components undergo congruent structural changes or mutual adaptation (Maturana and Verden-Zoller 2008, p. 169). Thus structural coupling concepts are central to both Anthropocene governance and sustainability science and are the basis of placing ‘the biosphere’ at the centre of the three-dimensional governance diamond in Fig. 1.

### ‘Joined-up’ governance, ‘deliverology’, targets

New Labour in the UK called for “joined-up government” building on understandings of the ‘third way’ (Giddens 1998). New Labour’s ‘Better Government’ agenda aimed to reduce cost and improve quality and effectiveness of public services by ensuring different agencies worked together. Conceptually, joined-up government is appealing but partnerships often fail due to traditional hierarchical structures limiting cooperation, so that people from different organisations only gave *the impression of dancing together while actually standing still* (Mackie 1999). For examples of multiple agencies with stakeholdings in water failing to form genuine partnerships see these authors: in England and Wales Collins et al. 2005; Collins and Ison 2010; Ison et al. 2004, and Australia Wallis and Ison 2011.

In the UK ‘joined-up government’ became an empty cliché, due to institutionalised settings that did not support enactment. These factors undermined New Labour’s espoused intentions, and ‘joined-up’ government failed to become conserved as a discourse coalition (Hajer 2009). In contrast, the ideology of targets and ‘deliverology’ became deeply entrenched, with both defining attributes of the new public management paradigm that infected corporations and government agencies (see McLoughlin et al. 2002; Straw 2014). With New Labour’s commitments to ‘deliverology’ (Barber et al. 2010) the ‘targets culture’ became endemic privileging systematic approaches over systemic ones, at considerable social cost (Seddon 2008; O’Donovan 2014; Pell 2012). For example, Caulkin (2009) observed that:

“pursuing targets to the detriment of patient care may have caused the deaths of 400 people at Stafford between 2005 and 2008 …. Put abstractly, targets distort judgment, disenfranchise professionals and wreck morale. Put concretely, in services where lives are at stake – as in the NHS or child protection – targets kill…target-driven organisations are institutionally witless because they face the wrong way: towards ministers and target-setters, not customers or citizens. Accusing them of neglecting customers to focus on targets … is like berating cats for eating small birds. That’s what they do”.

Caulkin’s (2009) analogy of “berating cats for eating small birds” to explain how organisational targets operate, exemplifies what we mean by a structure determined system—a cat does what it does because it is ‘structured’ by its evolutionary history.

In the UK and Australia, targets and ‘deliverology’ infused government practice competing with political discourses of ‘networked governance’, ‘public value’ and ‘joined-up government’ (Goldsmith and Eggers 2004; Kelly et al. 2002; MacDermott 2008; Mackie 1999, 2008). Targets were a dominant framing of the Murray Darling Basin Authority (MDBA) created to implement a further stage of Australia’s water reforms (Marshall and Alexandra 2016). The MDBA focused significant technical effort on setting targets for limiting water extraction—the legislated sustainable diversion limit (Ison et al. 2009; Alexandra 2017). At the time the MDBA was established a targets discourse was dominant within Australia. Prime Minister, Kevin Rudd waxed lyrical about programmatic specificity and the virtue of targets. The PM’s strategy had the hallmarks of ‘deliverology’ with Rudd saying that the public expects: “that we still deliver … we must have delivery as our core number one priority” (Oakes 2010, p. 82). While commitment to delivery may sound impressive, in its framing and prescriptions, it is a recipe for on-going systemic failure in the face of ‘wicked’ problems that require investment in social learning and ‘horizontal governance’ innovations i.e., developing cyber-systemic antidotes (Godden and Ison 2010; Ison 2007, 2010, 2017; Seddon 2008; Ison et al. 2013; Ison and Schlindwein 2015; Straw 2014).

### Reforming the governing of the Australian Murray-Darling River Basin (MDB)

#### The MDB—a microcosm of governing in the Anthropocene

**Fig. 3:**
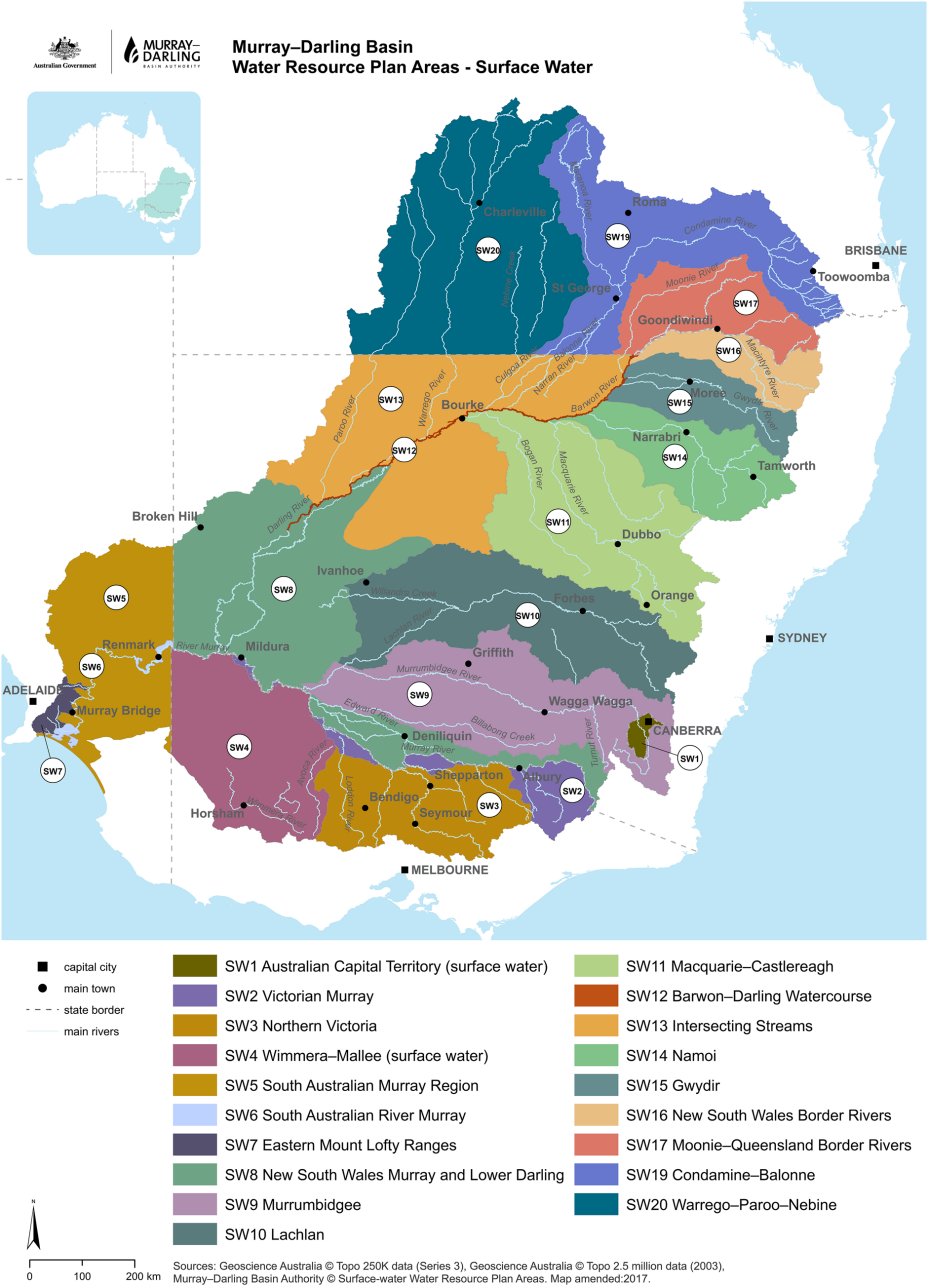
A map of the Australian Murray Darling Basin with planning regions as developed for the Murray-Darling Basin Plan. (Source: MDBA 2013)

Water policy reform in Australia’s largest and most economically important river basin, the Murray-Darling (Fig. 3) surfaces a multitude of issues. These range from the implications of climate change and the ‘death of stationarity’ (Milly et al. 2008) to multi-party governance under uncertainty (Alexandra 2017). It provides a useful microcosm of the challenges of governing in the Anthropocene because resetting the trajectories of large scale complex social-biophysical systems are inevitably constrained by institutional path dependence (Marshall and Alexandra 2016).

Faced with a severe drought, water over allocation, environmental deterioration, climate change, political discord and lack of action by the States, Australia’s Commonwealth government intervened in 2007. The Commonwealth abolished the Murray-Darling Basin Commission (MDBC) and established the Murray Darling Basin Authority (MDBA) (Alexandra 2017) that continues to face challenges arising from the complexity of transboundary basin governance (Armitage et al. 2015). Figure 3 illustrates some of this complexity.

The MDBA can be conceptualised as a hydro-social system coupled to its supra-socio-ecological system. Key system elements include (i) numerous State and Commonwealth statutes and ministries that influence the Basin (ii) the intergovernmental and transboundary coordination functions of the former MDBC and (iii) the biophysical system i.e., the rivers, water systems and water dependent ecosystems and (iv) and the industries and communities within the Basin all of which have their own structures and processes of political representation.

These elements were immersed in dominant discourses that help to frame and constrain policy options. For the MDBA these discourses could be defined as (i) Australia’s formally agreed water policies; (ii) national and international discourses concerning Integrated Water Resources Management (IWRM), governance innovation and effectiveness (e.g. joined-up government) and wicked problems; (iii) the Millennium drought ‘crisis’ narratives of water governance failures; (iv) the *Water Act 2007* shifting power from the States to the Commonwealth; (v) ruralism, rural decline and the need for rural development; (vi) public concern about environmental degradation; (vii) and matters arising from specific modes of employment, leadership and practice. We now explore some of the systemic issues arising from this constellation of factors.

#### The MDB initiative—empowerment and polarising discourse coalitions

From the late 1980s to the 2000’s the MDB Ministerial Council and MDBC advocated sustainable natural resources management (NRM) espousing community empowerment principles underpinning social movements like Landcare (Campbell 1994, 2010). These aligned with global sustainable development discourses, and emphasised partnering with communities in transitioning to more sustainable resource management (Alexandra and Riddington 2007). The MDB Initiative gave intergovernmental endorsement to Australian governments’ intentions to address sustainability. While historic roles of water sharing between states and operating the River Murray continued, the Initiative legitimised and resourced strategies and coalitions focused on integrated catchment management, linking land, water, governments and communities within an overarching framework of cooperative federalism (Connell 2007; Reeve et al. 2002). Basin governments formally shared power through the Ministerial Council’s consensus decision-making processes where member governments were deemed equal (Connell 2007).

Early in the twenty-first century, in the context of the severe Millennium drought (1996–2010) new ‘crisis’ narratives emerged. Powerful figures in government, industry and the media called for ‘Drought proofing’ and engineering regained its preeminent status in offering water ‘solutions’ (Crase et al. 2009). A narrative also arose that cooperative federalism’s consensus model for the MDB had failed, warranting Commonwealth interventions, imposing of stricter controls over the Basin States to save the Basin from an environmental ‘crisis’. In 2007 the national government intervened at a time when it was losing popularity with voters and desperately needed to be seen to be decisive on pressing national policy initiatives.

This dramatic swing in less than two decades was from a dominant discourse focused on sustainability through empowerment, cooperation and partnerships to one focused on fixing a narrowly defined problem—reallocating water shares between extractive and environmental use (Marshall and Alexandra 2016); in terms of ‘wicked’ and ‘tame’ framing the shift was more towards the latter. This fundamental shift can be interpreted through analysis of the meta-narratives about preferred roles of government and modes of governing. In the former approach, governance in complex federations requires focusing on soft power—influence, information, coordination, empowerment, etc—while in the latter the emphasis is on regulatory and legislative powers and their enforcement coupled with the provision of financial resources or ‘bribes’ to buy change (ibid).

Concerns emerged soon after the Commonwealth’s intervention disrupted the trusted relationships needed to negotiate across the multi sector, multi-scaled parties involved in governing the Basin’s natural resources (Ison et al. 2009; Curtis et al. 2014; Marshall and Smith 2010). Attempts to centralise powers concerned advocates of poly-centric governance (Marshall 2009). The newly formed MDBA also did not either communicate clearly about the purpose of the new organisation (Ison et al. 2009) or seek to establish a discourse coalition in support of the reform agenda (Marshall and Alexandra 2016).

Despite rhetorical support for ‘localism’ and community involvement in policy development (see for example Rudd 2008) surprisingly little was actually invested in supporting the co-production of reform solutions (Alston and Whittenbury 2011) or in enhancing the natural constituency for the reforms—those rural leaders already committed to sustainable natural resource management (NRM) (Campbell 2010). During the Plan’s development the reforms were consistently framed as ‘environment versus industry’, with powerful opponents of the reforms successfully influencing public opinion and policy options (Marshall and Alexandra 2016). Furthermore, during the early stages of the Plan’s development limited public involvement constrained the emergence of pro-reform advocates from the rural, environment or community sector. Processes of community empowerment were limited and sustainable NRM institutions and champions in Basin communities—like Landcare and Catchment groups—were not supported to become involved in co-designing reforms affecting their regions (Campbell 2010; Alston and Whittenbury 2011).The MDBA focused largely on the technical dimensions of target setting for the Plan, drawing heavily on hydrological science and modelling (Alexandra 2017). Insufficient attention to discursive and relational aspects of the reform process almost led to the Plan’s demise. Surprisingly, it can be argued that the reforms were rescued by vociferous public protests including burning of the ‘Guide to the Proposed Basin Plan’. These protests legitimised concerns of those directly affected who perceived significant negative impacts on their communities (Alston and Whittenbury 2011; Marshall and Alexandra 2016). This demonstrates that governments cannot expect to deliver reforms based on experts alone no matter how well researched the targets. To ignore the need for genuine citizen participation in co-production of reforms is to ignore the lessons of history (e.g. Curtis et al. 2014; Head 2011).

Unfortunately, community involvement in co-production of reforms rarely occurred in developing the Basin Plan (Campbell 2010; Alston and Whittenbury 2011); instead the MDBA entrenched a technocratic target setting paradigm, exemplifying a mismatch between espoused theory and theory-in-use (Argyris and Schön 1974). In many ‘wicked problem’ situations it is likely that the governance dynamics conform to this mismatch, and will continue to do so unless there are fundamental changes in governance practices. Ironically this is what the APSC (2007a) advocated in the year before the MDBA was established.

## Discussion

This paper illuminates governance deficits when the exigencies of the Anthropocene are upon us (see Steffan et al. 2007). Insights are provided into the enduring divide between policy rhetoric and praxis occurring under constraining institutional arrangements. Our inquiry draws on water governance reforms noting that water is one of many issues demanding transformative governing in the Anthropocene (Rockström et al. 2014).

### Purposeful governing that maintains structural coupling?

In terms of structural coupling both cases highlight systemic failings in the ‘governance social system’, especially in terms of the mismatch between espoused purpose (e.g. joined up governing) and realised practice (Straw 2014). For the MDB, evidence of systemic failings emerged in 2017 in the state of NSW (New South Wales) prompting an independent review (Matthews 2017). The principal finding was that water-related compliance and enforcement arrangements were ineffectual, requiring significant and urgent improvement, including more transparency, and more effective enactment of compliance roles (ibid).

If understood through the conceptual lens of structural determinism then four possibilities for structural change in a system arise (Maturana and Verden-Zoller 2008, p. 165): (i) changes of state—changes to the internal structural dynamics of the system i.e., change from within; (ii) disintegrations, arising from internal structural changes which lead to loss of organization of the system; (iii) perturbations, changes triggered in the system by external agents but which maintain conservation of the system and (iv) destructive interactions, structural changes triggered by external agents such that the system disintegrates.

Context specific research is needed to better appreciate this set of change possibilities for governance innovation. What is clear is that attempts in the UK to introduce and build discourse coalitions around ‘joined-up government’ failed. As did attempts by the APSC to do the same around governing ‘wicked problems’. Our cases suggest that strategies (i) and (iii) above were not very effective. In systemic terms, structural determinism can be understood as emerging from the autonomy and closure of the system, and if purpose is what a system does, then it is apparent that we live within a crisis of social purpose (see Fig. 1). Our cases suggest governance systems with structures that fail to absorb within themselves the emerging complexity that generates the Anthropocene.

Analyses by Kelly (2014) and Tingle (2015) suggest that change strategy (ii) may be unfolding in Australia, with few obvious strategies or innovations emerging to break out of this trajectory. In the UK, Straw’s (2014) proposals offer the possibility of creating a new discourse coalition though in many ways it exemplifies change strategy (iv) of picking up the pieces, after destructive disintegration. Unfortunately demands for the purposeful demise and replacement of current governance systems are not yet well formulated, hence Fig. 1. However, it should not be forgotten that the purposeful and peaceful design of novel governance systems by citizens has been achieved in the past, e.g. New Zealand, Australia, USA, South Africa etc.

In terms of cyber-systemic praxes the most apparent failings arising from the cases are (i) situational framing failure; (ii) creating systematic rather than systemic initial starting conditions; (iii) emphasising and using institutions (targets) with systematic as opposed to systemic affordances, and (iv) imposing a blueprint onto the situation rather than being open to multiple, partial perspectives in processes of systemic co-inquiry and co-design, e.g. empowerment and co-production strategies as developed in Australian NRM (Campbell 2010).

On the last point, our research across many domains, in a range of countries provides evidence that capacity and capability to enact cyber-systemic alternatives can be facilitated with moderate investment. However, evidence is emerging that in the absence of a convivial governance system individual, institutional or praxis innovations are insufficient. Individual innovations are not the antidotes to the malaise of modern governments nor the basis for effectively governing in the Anthropocene (e.g. Pollard and Toit 2011; Mackay et al. 2014; Ison 2016a, b; 2017). However there is recognition that these approaches need to be institutionally adopted. For example, in 2009 the then APS Commissioner advocated that:

“Tackling these [wicked] problems will require new ways of thinking, including systems thinking—grasping the big picture; analysing interrelationships and comprehending ‘messy’ situations with multiple, overlapping perspectives” (Briggs 2009).

She advocated adopting “new modes of policy implementation*”* using new capabilities including:

- System thinking, problem framing and boundary setting
- fresh thinking on intractable problems
- collaboration across organisational and disciplinary boundaries
- working in situations characterised by high levels of uncertainty
- being able to tolerate rapid change in problem definition
- engaging stakeholders as joint decision-makers (not just providers or recipients of services)

This formulation was insufficient because processes of institutionalisation remain weak and the mainstream, systematic, paradigm consistently reasserts itself. Recent government reforms create little confidence that supportive institutional arrangements are arising to enable effective governing in the Anthropocene (Curtin 2014; Ison 2010; Jasanoff 2010). Persistent policy failures demonstrate that much contemporary public sector governing has flawed foundations (Seddon 2014) and inadequate capacity for change (Ison 2016b).

### Governance reform—emergent or ‘muddling through’?

In 2012 the MDB Plan was gazetted as a regulatory instrument in the Australian Parliament. The MDBA muddled through, with the Plan providing a milestone in “this complex, messy and, at times, irrational reform process” (Skinner and Langford 2013, p. 871). Lindblom (1959) wrote in favour of ‘muddling through’ rather than unquestioning adherence to specific policy techniques and methods. While clumsy solutions embracing multiple perspectives and different logics are generally preferable to standardized approaches to policy formulation (Ingram 2008, p. 17) is ‘muddling through’ enough in the Anthropocene?

Ingram (2008) argues that there are no universal remedies for good governance but emphasises the importance of contextually relevant design of policies and practices. Contextual design, however, requires effective praxis within an enabling environment (Ison et al. 2014b; Metcalf 2014). Fairtlough (2007) posits three modes of operating in organisational life; hierarchy is the most common, virtually hegemonic, but hierarchical or command and control approaches are poorly suited to a climate-changing world (Alexandra 2012). Fairtlough’s second category is heterarchy, comprising a balance of powers rather than a single rule through hierarchy (e.g. partners in a law firm or the MDBC model). Heterarchical modes include mutual societies or cooperatives, community climate coalitions or irrigation cooperatives. Fairtlough’s third category is ‘responsible autonomy’ in which individuals or groups make decisions yet are accountable for their outcomes. Landcare groups as originally conceived in Australia exemplified ‘responsible autonomy’ (Campbell 1994) but with increasing appropriation by central government they have suffered from hierarchical strictures (Robins and Kanowski 2011).

Cyber-systems scholars understand that control can be achieved through processes of self-organization (or responsible autonomy) and that this control differs from that achieved through hierarchy. Hierarchical command and control and linear communication models are ill suited to governance in the Anthropocene (Ison 2017) and are prone to failure if they lack distributed, localised variety for responding to and managing emergent possibilities. Seen from this perspective what might appear as ‘muddling through’ could be the realisation of emergent patterns and configurations of bottom-up innovation based on the valuing of differences.

Transformational innovations are clearly needed to break free from the constraints of historically- generated structure determined systems (Fig. 1), leading to several questions: First, what contributes to governance innovation and how can we purposefully create the conditions for self-organisation—a key attribute of cyber-systemic, adaptive governance? Second, will Anthropocene societies demand governance models and institutional arrangements conducive to systemic governing? Third, if so the next question is what to do? Ingram et al. (2014) suggest enhancing *“emergent, alternative coalitions that challenge the status quo”* using *“narrative-networks* [on the] *fringe of the extant power structure*.” This is an expansion of ‘discourse coalitions’, but we go further and argue the need to foster innovative institutions and praxis coalitions (Mackay et al. 2014; Ison et al. 2011; Ayre and Nettle 2015) who redesign the institutionalised structures of governance. That is, they engage in cyber-systemic design of new structural configurations that offer affordances to alternative discourses and enable cyber-systemic governing praxes.

### Implications for sustainability science

The analysis provided in this paper has a number of implications for the practices of sustainability science. First, by offering explicit models of governing as steering it articulates the intensive demands for information as feedback, which sustainability science can deliver if its coupled within the social governing system and is timely rather than attenuated. Second, by recognising governing as dynamic and relational, it emphasises processes and partnerships (co-design), not the separation of policy and science as distinct domains. Third by drawing attention to ideas of enactment and praxis (theory informed practice) it calls for greater systemic sensibility and literacy on the part of those who practice science and governing. Finally, there are many opportunities for sustainability scientists to engage with questions of how modes and structures of governing (governments and governance) frame science and the priorities for research and how to conduct research into the central questions of this paper: how to design or inform the design of governance institutions suited to more cyber-systemic governing in the Anthropocene.

## Conclusions

An antidote is something that prevents or counteracts injurious or unwanted effects. To the extent that our cases depict features that constitute a malaise of modern governance there is a clear need for antidotes, however, as always, diagnosis is required before prescription. This analysis is offered in the spirit of improving governing as practiced, and while encouraging innovations we do not seek to prescribe any universal remedies, for to do so would inappropriately tame a ‘wicked problem’ and ignore the need for novel co-designing or co-inquiry based on contextually rich local variety (Foster et al. 2016). Neither do we wish to simplify the solutions as requiring either cultural change or structural change—we are advocating both.

Public sector agencies with their traditions and structures are deeply hierarchical; staff typically experience government organisations as strongly held within a culture of command-and-control. Head and Alford (2014) demonstrate that “efforts to deal with wicked problems are impeded by the working mechanisms of the public sector—its characteristic ways of making decisions, organizing, financing, staffing, and controlling”. In contrast what is needed, they argue, (ibid) are “strategies for dealing with wicked problems under these governmental and administrative constraints—such as going beyond technical/rational thinking, collaborative working, new modes of leadership, and reforming the managerial infrastructure of government.” While these are important ingredients of transforming the public sector they neglect the design and introduction of institutions conducive to praxis innovation, and therefore questions of significant structural reform as depicted in Fig. 1.

As Fox et al. (2017) have shown a changing political or legal context helps create space for assertion of novel ways of knowing that are also new ways of doing. Their work, asserting “indigenous spiritual and cultural values” whilst ‘repair[ing] community relationships with water [to] empower communities vis-à-vis the wider society’ (p. 1), elucidates our perspective on the potential of cyber-systemic ways of knowing and acting to reframe understandings of the coupling of Anthropocene governance with sustainability science. For governing in the Anthropocene we claim there are cyber-systemic antidotes to the malaise of modern governance. We foreshadow the emergence of governance design logics that bring new dimensions to policy development (Bason 2014; Ison 2016a) including active framing choices, and institutional and other innovations that break the current structural determinism of our governance systems.

## Footnotes

1 For us the naming of a system is an epistemological framing that in practical terms invites reflection on the question: can new understanding, practice or transformation be effected if I/we name, see, or bring forth system *X* or *Y* (etc) in a situation of concern? Our preferred starting point is not to see systems as having some pre-existing ontological status (see Ison 2017).

2 See:http://www.sympoetic.net/Systems/structural_determinism.html Accessed 4th May 2017.
